# Autopolyploidy, Allopolyploidy, and Phylogenetic Networks with Horizontal Arcs

**DOI:** 10.1007/s11538-023-01140-9

**Published:** 2023-04-06

**Authors:** K. T. Huber, L. J. Maher

**Affiliations:** grid.8273.e 0000 0001 1092 7967 UEA, Norwich, UK

**Keywords:** Phylogenetic network, Ploidy profile, Polyploid phylogenetics, Ploidy profile space

## Abstract

Polyploidization is an evolutionary process by which a species acquires multiple copies of its complete set of chromosomes. The reticulate nature of the signal left behind by it means that phylogenetic networks offer themselves as a framework to reconstruct the evolutionary past of species affected by it. The main strategy for doing this is to first construct a so-called multiple-labelled tree and to then somehow derive such a network from it. The following question therefore arises: How much can be said about that past if such a tree is not readily available? By viewing a polyploid dataset as a certain vector which we call a ploidy (level) profile, we show that among other results, there always exists a phylogenetic network in the form of a beaded phylogenetic tree with additional arcs that realizes a given ploidy profile. Intriguingly, the two end vertices of almost all of these additional arcs can be interpreted as having co-existed in time thereby adding biological realism to our network, a feature that is, in general, not enjoyed by phylogenetic networks. In addition, we show that our network may be viewed as a generator of ploidy profile space, a novel concept similar to phylogenetic tree space that we introduce to be able to compare phylogenetic networks that realize one and the same ploidy profile. We illustrate our findings in terms of a publicly available Viola dataset.

## Introduction

Polyploidization is an evolutionary phenomenon thought to be one of the key players in plant evolution. It has, however, also been observed in fish (Leggatt and Iwama 2003) and fungi (Albertin and Marullo 2012) and arises when a species acquires multiple copies of its full set of chromosomes. This can be the result of, for example, a species undergoing whole genome duplication (autopolyploidization) or through acquisition of a further complete set of chromosomes via interbreeding with a different, usually closely related, species (allopolyploidization) (Albertin and Marullo 2012) (see also Doyle and Sherman-Broyles 2017 who point out that the definitions of allopolyploidy and autopolyploidy are controversial). Examples of autopolyploids include crop potato (The Potato Sequencing Consortium 2011) and bananas and watermelon (Vaoquaux et al. 2000), and examples of allopolyploids include bread wheat (Marcussen et al. 2014) and oilseed rape. Understanding better how polyploids have arisen (and still arise) therefore has potentially far reaching consequences.

Many tools for shedding light into the evolutionary past of a polyploid data set such as PADRE (Lott et al. 2009) and AlloPPnet (Jones et al. 2013) start with a multiple-labelled tree, sometimes also called a MUL-tree or a multi-labelled tree.

**Fig. 1:**
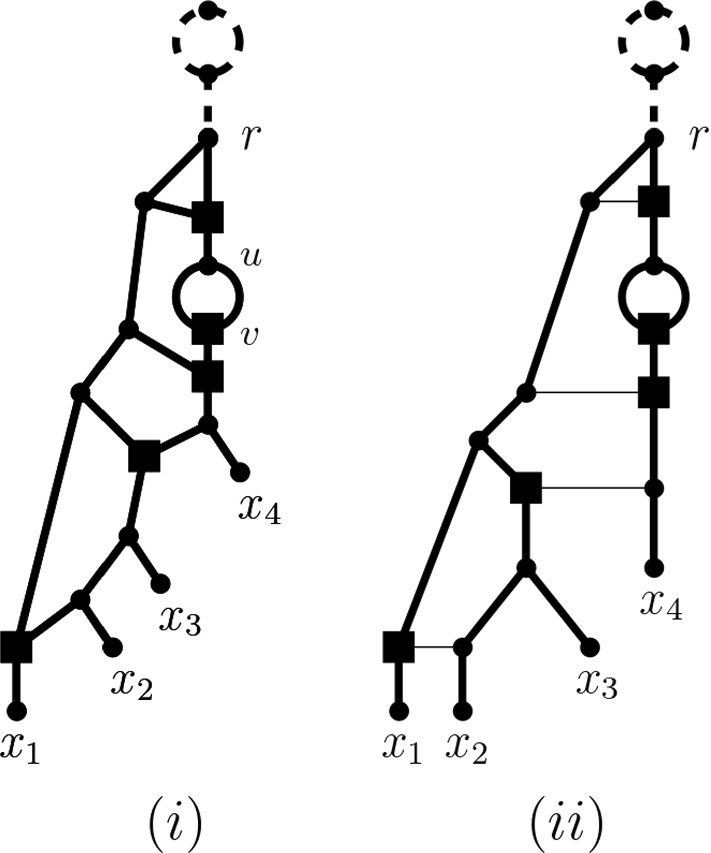
(i) One of potentially many phylogenetic networks that realize the ploidy levels 14, 12, 12, 10 of a set $X=\{x_1,x_2,x_3,x_4\}$ of taxa where 14 is the ploidy level of $x_1$, the ploidy level of $x_2$ and $x_3$ is 12, respectively, and the ploidy level of $x_4$ is 10. To improve clarity of exposition, we always assume that unless indicated otherwise, arcs are directed away from the root (which is always at the top). (ii) The network in (i) represented in such a way that every reticulation vertex (indicated throughout the paper by a square and defined below) has precisely one incoming horizontal arc implying that the end vertices of such an arc represent ancestral species that have existed at the same point in time. In both (i) and (ii), the phylogenetic network resulting from deleting the dashed bead and its dashed outgoing arc realizes the ploidy profile $\textbf{m}=(7, 6, 6, 5)$

These types of trees differ from the standard phylogenetic trees by allowing two or more leaves to be labelled with the same species. In the case of PADRE, a (phylogenetic) network is then produced from such a tree by folding it up as described in, for example, Huber et al. (2006). Referring the interested reader to Fig. 1(i) for an example and below for definitions, it suffices to say at this stage that a phylogenetic network is a directed graph with leaf set, a set of taxa (e.g. species) of interest, a single root (usually drawn at the top), and no directed cycles. Note that to be able to account for autopolyploidy, we deviate from the standard definition of a phylogenetic network (see e.g. Steel 2016) by also allowing it to contain *beads*, that is, pairs of parallel arcs, as is the case in the networks depicted in Fig. 1. Polyploidization events are represented in such networks as *reticulation vertices*, that is, vertices with more than one arc coming into them. For clarity of exposition, we indicate reticulation vertices throughout the paper in terms of squares. Although PADRE is generally fast and not constrained by an upper limit on the ploidy levels in a dataset of interest, its underlying assumptions imply that it is highly susceptible to noise in the multiple-labelled tree from which the network is constructed. In the case of AlloPPnet, a phylogenetic network is inferred using, among other techniques, the multispecies coalescent to account for incomplete lineage sorting. The computational demands of AlloPPnet however mean that it can only be applied on relatively small data sets that contain only diploids and tetraploids (Rothfels 2021).

One approach to obtain an input multiple-labelled tree for PADRE is to try and construct it as a consensus multiple-labelled tree from a set of multiple-labelled gene trees. This task is relatively straightforward for phylogenetic trees by applying, for example, some kind of consensus approach to the collection of clusters induced by the trees. The corresponding approach for constructing a consensus multiple-labelled tree from a collection of multiple-labelled gene trees however gives rise to a computationally hard decision problem (Huber et al. 2009). A consensus multiple-labelled tree might therefore not always be readily available for a dataset. The following question therefore arises: How much can we say about the reticulate evolutionary past of a polyploid dataset if a multiple-labelled tree is not readily available? Since one of the signatures left by polyploidization is the *ploidy level* of a species (i.e. the number of copies of the complete set of chromosomes of that species), we address this question in terms of a dataset’s ploidy levels or more precisely the ploidy levels of the taxa that make up the dataset using phylogenetic networks as a framework. Interpreting the ploidy level of a species as the number of directed paths from the root of a phylogenetic network *N* to the leaf in *N* that represents that species, Fig. 1(i) implies that in general, ploidy levels do not preserve the topology of the phylogenetic network that induced them. For example, swapping $x_2$ with $x_3$ in that network results in a phylogenetic network that induces the same ploidy levels on $\{x_1,\ldots , x_4\}$ as the network pictured in Fig. 1(i). We are therefore interested in understanding to what extent a phylogenetic network representing the evolutionary past of a polyploid dataset can be derived solely from the ploidy levels of the species that make up the dataset.

Note that since polyploidization events are assumed to be rare, we are particularly interested in phylogenetic networks that enjoy this property and also aim to minimize the number of reticulation vertices. From the perspective of reducing the complexity of our mathematical arguments, this immediately implies that we may assume the ploidy level of a taxon to not be even. Indeed, if we have a dataset where every ploidy level is of the form $m=2m'$, some positive integer $m'$, then since polyploidization events are assumed to be rare, we may assume the last common ancestor of the dataset’s taxa to have undergone autopolyploidization. The root of a phylogenetic network *N* that represents the evolutionary past of the dataset is therefore contained in a bead and that bead accounts for the factor 2 in *m*. Thus, the phylogenetic network obtained by removing this bead and the arc that joins it to the rest of *N* is a phylogenetic network that represents the factor $m'$ of *m* in terms of numbers of directed paths from the root to the leaves.

In view of the above, we call any (finite) vector of positive integers that is indexed by a (finite, non-empty) set *X* a *ploidy profile (on* *X*). Although related to the recently introduced *ancestral profiles* (Erdös et al. 2019) (but also see (Bai et al. 2021)), ploidy profiles differ from them by only recording the number of directed paths from the root of a phylogenetic network *N* to every leaf of *N*. Ancestral profiles on the other hand record the number of directed paths from every non-leaf vertex in *N* to all the leaves below that vertex. In particular, a ploidy profile is an element of an ancestral profile of a phylogenetic network.

To help motivate our approach for addressing our question, consider the phylogenetic network *N* with leaf set $X=\{x_1,x_2,x_3,x_4\}$ depicted in Fig. 1(i) where the square vertices at the end of each pair of two parallel arcs represent autopolyploidization and the remaining four square vertices represent allopolyploidization. Then taking for each taxon *x* in *X*, the number of directed paths from the root of the network to *x* results in the ploidy profile $\textbf{m}=(14,12,12,10)$ where the first component is indexed by $x_1$, the second by $x_2$ and so on. Each component in $\textbf{m}$ is of the form 2*m*, some positive integer *m*, and the phylogenetic network rooted at *r* obtained by removing the dashed bead together with the dashed arc coming into *r* represents the ploidy profile $\mathbf {m'}=(7,6,6,5)$ in terms of numbers of directed paths from *r* to the leaves. With this in mind, we say that a ploidy profile $\textbf{m}=(m_1,\ldots , m_n)$, $n\ge 1$ on $X=\{x_1,\ldots , x_n\}$ is *realized* by a phylogenetic network *N* with leaf set *X* if, for all $1\le i\le n$, the number of directed paths from the root of *N* to $x_i$ is $m_i$. For example, both phylogenetic networks pictured in Fig. 1 realize the ploidy profile $\textbf{m} = (14,12,12,10)$ indexed by $X = \{x_1,x_2,x_3,x_4\}$.

Contributing to the emerging field of *Polyploid Phylogenetics* (Rothfels 2021), a first inroad into our question was made in Huber and Maher (2022) by studying the hybrid number of a ploidy profile $\textbf{m}$, that is, the minimal number of polyploidization events required by a phylogenetic network to realize $\textbf{m}$. As it turns out, the arguments underlying the results in Huber and Maher (2022) largely rely on a certain iteratively constructed network that realizes $\textbf{m}$. Denoting for a choice *C* of initializing network the generated network by $N(\textbf{m})=N_C(\textbf{m})$ and changing the network initializing that construction in a way that does not affect the main findings in Huber and Maher (2022) (see below for details), we show that even more can be said about ploidy profiles. For example, our first result (Proposition 1) shows that $N(\textbf{m})$ may be thought of as a generator of ploidy profile space (defined in a similar way as phylogenetic tree space) in the sense that any realization of $\textbf{m}$ can be reached from $N(\textbf{m})$ via a number of multiple-labelled tree editing operations and reticulation vertex splitting operations. As an immediate consequence of this, we obtain a distance measure for phylogenetic networks that realize one and the same ploidy profile. On a more speculative level, it might be interesting to see if $N(\textbf{m})$ lends itself as a useful prior for a Bayesian method along the lines as described in van Iersel et al. (2022).

Our second result (Theorem 2) shows that a key concept introduced in Huber and Maher (2022) called the simplification sequence of a ploidy profile $\textbf{m}$ is in fact closely related to the notion of a cherry reduction sequence (Erdös et al. 2019) for $N(\textbf{m})$, also called a cherry picking sequence in Janssen and Murakami (2021). In case autopolyploidy is not suspected to have played a role in the evolution of a dataset, this implies that the network $N(\textbf{m})$ can also be reconstructed from phylogenetic networks on three leaves called trinets (Semple and Toft 2021). These can be obtained from a dataset using, for example, the TriLoNet software (Oldman et al. 2021).

Exemplified in terms of the phylogenetic network depicted in Fig. 1(ii) for the ploidy profile (14, 12, 12, 10), our third result (Theorem 3) implies that for any ploidy profile, we can always find a phylogenetic network realizing it in the form of a phylogenetic tree that potentially contains beads to which additional arcs have been added, and at most, one of those arcs is not horizontal. In the context of this, it is important to note that in general, a phylogenetic network cannot be thought of as a phylogenetic tree with additional arcs let alone horizontal ones. The reason for this is that horizontal arcs imply that the ancestral taxa joined by such an arc must have existed at the same time (see also Steel 2016, Section 10.3.3) for more on this and the Viola dataset below for an example).

The remainder of the paper is organized as follows. In the next section, we review relevant basic terminology surrounding graphs, phylogenetic networks and ploidy profiles. For a ploidy profile $\textbf{m}$, we outline the construction of the network $N(\textbf{m})$ in Sect. 3. This includes the definition of the simplification sequence for $\textbf{m}$. Subsequent to this, we introduce ploidy profile space in Sect. 4 and also establish Proposition 1 in that section. Sections 6 is concerned with establishing Theorems 2 and 3. To do this, we use Theorem 1 which we establish in Sect. 5. That theorem is underpinned by the concept of a so-called HGT-consistent labelling introduced in van Iersel et al. (2021), a notion that we extend to our types of phylogenetic networks here. In the last but one section, we employ a simplified version of a Viola dataset from Marcussen et al. (2012) to help explain our findings within the context of a real biological dataset. We conclude with some potential directions of further research in the last section.

## Preliminaries

We start with introducing basic concepts surrounding phylogenetic networks.

Throughout the paper, we assume that *X* is a (finite) set that contains at least one element. Also, we denote the number of elements in *X* by *n*.

### Graphs

Suppose for the following that *G* is a directed acyclic graph with a single root which might contain parallel arcs but no loops.

We denote an arc starting at a vertex *u* and ending in a vertex *v* by (*u*, *v*). If there exist two arcs from *u* to *v*, then we refer to the pair of arcs from *u* to *v* as a *bead* of *G*.

Suppose *v* is a vertex of *G*. Then, we refer to the number of arcs coming into *v* as the *indegree* of *v* in *G* and denote it by ${\textit{indeg}}(v)$. Similarly, we call the number of outgoing arcs of *v* the *outdegree* of *v* in *G* and denote it by ${\textit{outdeg}}(v)$. We call *v* the *root* of *G*, if ${\textit{indeg}}(v)=0$, and we call *v* a *leaf* of *G* if ${\textit{indeg}}(v)=1$ and ${\textit{outdeg}}(v)=0$. We denote the set of vertices of *G* by *V*(*G*) and the set of leaves of *G* by *L*(*G*). We call *v* a *tree vertex* if ${\textit{outdeg}}(v)=2$ and ${\textit{indeg}}(v)=1$, and we call *v* a *reticulation vertex* if ${\textit{indeg}}(v)=2$ and ${\textit{outdeg}}(v)=1$.

If *w* is also a vertex in *G*, then we say that *w* is *below* *v* if either $v=w$ or there exists a directed path from the root of *G* to *w* that crosses *v*. If *w* is below *v* and $v\not =w$, then we say that *w* is *strictly below* *v*. A *parent* of a vertex *v* is the vertex connected to *v* on the path to the root. A *child* of a vertex *v* is a vertex of which *v* is the parent.

Suppose *a* and *b* are two distinct leaves of *G*. Then, the set $\{a,b\}$ is called a *cherry* of *G* if the parent $p_a$ of *a* is also the parent of *b*. If the parent $p_b$ of *b* is a reticulation vertex and there is an arc $(p_a,p_b)$ from $p_a$ to $p_b$, then the set $\{a,b\}$ is called a *reticulate cherry*. In this case, the arc $(p_a,p_b)$ is called a *reticulation arc* of *G*, and the leaf *b* is called a *reticulation leaf* of *G*.

For example, $x_1$ is the reticulate leaf of the reticulate cherry $\{x_1,x_2\}$ in the graph depicted in Fig. 1(i). The parent of $x_2$ is a tree vertex, and the parent of $x_1$ is a reticulation vertex. The vertices *u* and *v* form a bead.

### Phylogenetic Networks and Trees

Suppose *G* is a graph as described above. If *G* contains at least three vertices, then we call *G* a *(phylogenetic) network (on* *X*) if the outdegree of the root $\rho$ of *G* is 2, the leaf set of *G* is *X*, and every vertex other than $\rho$ or a leaf is a tree vertex or a reticulation vertex. Note that our definition of a phylogenetic network differs from the standard definition of such an object (see e.g. Steel 2016) by allowing the network to contain beads and *X* to contain a single element. To distinguish between our type of phylogenetic networks and the standard type of phylogenetic networks, we refer to the latter as *beadless* phylogenetic networks. We call a phylogenetic network (on *X*) a *phylogenetic tree (on* *X*) if it does not contain any reticulation vertices.

Finally, we call a phylogenetic network *N* on *X* such that *N* is either a phylogenetic tree on *X* or every reticulation vertex of *N* is contained in a bead a *beaded tree* (see e.g. van Iersel et al. 2018 and Huber et al. 2021 for more on such graphs).

### Ploidy Profiles

Let $X=\{x_1,\ldots , x_n\}$. Then, as mentioned in the introduction, a *ploidy profile* $\textbf{m}=(m_1,\ldots , m_n)$ (*on* *X*) is an *n*-dimensional vector of positive integers such that each component is indexed by an element in *X*. For ease of readability, we will assume from now on that the elements in *X* are always ordered in such a way that $x_i$ indexes component $m_i$ of $\textbf{m}$, for all $1\le i\le n$, and that $\textbf{m}$ is in *descending order*, that is, $m_i\ge m_{i+1}$ holds for all $1\le i\le n-1$. For example, the vector $\textbf{m}=(7,6,6,5)$ is a ploidy profile on $X=\{x_1,x_2.x_3,x_4\}$ where $x_1$ indexes the first component, i.e. 7, $x_2$ indexes the second component, and so on.

Suppose $\textbf{m}=(m_1,\ldots , m_n)$ is a ploidy profile on *X*. If $m_1\ge 2$ and all other components of $\textbf{m}$ are 1, then we call $\textbf{m}$ a *simple* ploidy profile. If $\textbf{m}$ is a simple ploidy profile and $n=1$, then we call $\textbf{m}$ a *strictly simple* ploidy profile. Finally, we say that a phylogenetic network is a *realization* of $\textbf{m}$ if it realizes $\textbf{m}$ (as defined in the introduction). For example, the ploidy profile $\textbf{m}=(77,1,1,1)$ is simple but not strictly simple and the ploidy profile (77) is strictly simple. The phylogenetic networks shown in Fig. 1 are realizations of the ploidy profile $\textbf{m} = (14,12,12,10)$.

## Realizing Ploidy Profiles

We start this section by introducing further terminology which will allow us to construct our network $N(\textbf{m})$ from a ploidy profile $\textbf{m}$. To avoid undesirable uniqueness issues, we remark that our construction is slightly different from the construction of the corresponding network for $\textbf{m}$ introduced in Huber and Maher (2022); in that we choose a different network with which we initialize its construction. As part of our construction, we also include a worked example at the end of this section.

Suppose $\textbf{m}=(m_1, m_2, \ldots , m_n)$ is a ploidy profile on $X=\{x_1, x_2, \ldots , x_n\}$. Then, we first construct a sequence $\sigma (\textbf{m})$ of ploidy profiles from $\textbf{m}$ which we call the *simplification sequence* for $\textbf{m}$. This sequence starts with the ploidy profile $\textbf{m}$ and terminates with a certain simple ploidy profile $\mathbf {m_t}$ which we call the *terminal element* of $\sigma (\textbf{m})$. If $\textbf{m}$ is simple, then we define $\sigma (\textbf{m})$ to contain only $\textbf{m}$. Thus, $\textbf{m}= \mathbf {m_t}$ in this case.

Assume for the following that $\textbf{m}$ is not simple. To define $\sigma (\textbf{m})$ in this case, assume furthermore that all ploidy profiles in $\sigma (\textbf{m})$ have been constructed already up to and including a ploidy profile $\mathbf {m'}=(m_1',\ldots , m_q')$ on some set $X'=\{x_1',\ldots , x_q'\}$, some $q\ge 1$. If $\mathbf {m'}$ is simple then we define $\mathbf {m'}$ to be $\mathbf {m_t}$. So assume that $\mathbf {m'}\not =\mathbf {m_t}$. Put $\alpha = m_1' - m_2'$. Let $X''$ denote the set that indexes the next ploidy profile in $\sigma (\textbf{m})$ which we call $\mathbf {m''}$. Then, $\mathbf {m''}$ and $X''$ are obtained from $\mathbf {m'}$ and $X'$ by applying one of the following cases.

- If $\alpha =0$, then delete $m_1'$ from $\mathbf {m'}$ and its index $x_1'$ from $X'$. To obtain $X''$, rename the elements $x_{k}'$ as $x_{k-1}''$, $2\le k\le q$.
- If $\alpha >m_2'$, then replace $m_1'$ by $\alpha$. The set $X''$ is $X'$ in this case and the indexing of the components of $\mathbf {m''}$ is as in $\mathbf {m'}$.
- If $\alpha \le m_2'$, then remove $m'_1$ from $\mathbf {m'}$ and its index $x_1'$ from $X'$ to obtain a ploidy profile $\textbf{a}$ on $X'-\{x'_1\}$. Into $\textbf{a}$, insert $\alpha$ so that the resulting integer vector $\textbf{b}$ is a ploidy profile on $X''=X'-\{x'_1\}\cup \{x\}$ where *x* is an element not already contained in $X'$. That element is used to index $\alpha$ in $\textbf{b}$. As $\textbf{a}$ might already contain a component with value $\alpha$, we also require that $\alpha$ is inserted into $\textbf{a}$ directly after the last occurrence of $\alpha$ to ensure that $\textbf{b}$ is unique. Next, relabel the elements in $X''$ so that the indexing of $\textbf{b}$ conforms to our indexing convention for ploidy profiles. Finally, put $\mathbf {m''}=\textbf{b}$.

This completes the construction of the simplification sequence for $\textbf{m}$. To aid intuition, we present the simplification sequence for the ploidy profile $\textbf{m}=(7,6,6,5)$ at the end of this section.

To obtain our realization for our ploidy profile $\textbf{m}$, we next choose a *core network* for $\textbf{m}$, that is, a phylogenetic network *N* that realizes the terminal element $\mathbf {m_t}$. This is always possible since for any simple ploidy profile $(m_1,\ldots , m_n)$ on $X=\{x_1,\ldots , x_n\}$ such a network can be obtained using the following naive approach. Take a directed path *P* with $n+2(m_1-1)$ vertices and label the first $m_1-1$ vertices on *P* by $v_i$, $1\le i\le m_1-1$ and the next $n-1$ vertices by $w_i$, $2\le i\le n$. Starting at the other end of *P*, label the first vertex $x_1$ and the remaining $m_1-1$ vertices by $v_i'$, $1\le i\le m_1-1$. Finally, for all $2\le i\le n$ attach the arc $(w_i,x_i)$ and, for all $1\le i\le m_1-1$, the arc $(v_i,v_i')$. By construction, the resulting graph is a phylogenetic network (without horizontal arcs) that realizes $\mathbf {m_t}$. To keep the description of the construction of $N(\textbf{m})$ from $N(\mathbf {m_t})$ compact, we refer the interested reader to Sect. 5 for the construction of a more attractive choice of core network for $\textbf{m}$.

Starting with a core network *N* for $\textbf{m}$, we then apply a traceback through $\sigma (\textbf{m})$ to obtain $N(\textbf{m})$ via the addition of vertices and arcs (see e.g. Janssen and Murakami 2021, where, in a different context, this process was called “adding” vertices). For this, we distinguish the same cases as before. More precisely, if $\textbf{m}$ is simple and therefore the terminal element of $\sigma (\textbf{m})$, we define $N(\textbf{m})$ to be *N*. So assume that $\textbf{m}$ is not simple and that, starting at $\mathbf {m_t}$, for all ploidy profiles in $\sigma (\textbf{m})$ up to and including a ploidy profile $\mathbf {m''}$ on $X''$ a realization of them has already been constructed. Let $N''$ denote the realization obtained for $\mathbf {m''}$. As before, let $\mathbf {m'}$ on $X'$ denote the ploidy profile in $\sigma (\textbf{m})$ that precedes $\mathbf {m''}$. For clarity of presentation of the main ideas, we remark that in each of the following cases, the set $X'$ is obtained from $X''$ by reversing the indexing that formed part of the corresponding case in the construction of $\sigma (\textbf{m})$.

- If $\alpha =0$, then replace $x_1''$ by the cherry $\{x_1',x_2'\}$.
- If $\alpha > m_2$, then subdivide the incoming arc of $x_1''$ by a new vertex *u*. Next, subdivide the incoming arc of $x_2''$ by a vertex *v* and add the new arc (*v*, *u*).
- If $\alpha \le m_2$, then let $\textbf{a}$ and $\textbf{b}$ be in the corresponding case of the construction of $\sigma (\textbf{m})$. Let *j* denote the index of the component of $\textbf{b}$ that was inserted into $\textbf{a}$ as part of the construction of $\textbf{b}$. Then, subdivide the incoming arc of $x_{j}''$ by a new vertex *v* and replace $x_1''$ by the cherry $\{x_1',x_2'\}$. Next, subdivide the incoming arc of $x_1'$ by a new vertex *u*. Finally, add the arc (*v*, *u*) and delete $x_j''$ and its incoming arc $(u,x_j'')$ (suppressing the resulting indegree and outdegree one vertex).

**Fig. 2:**
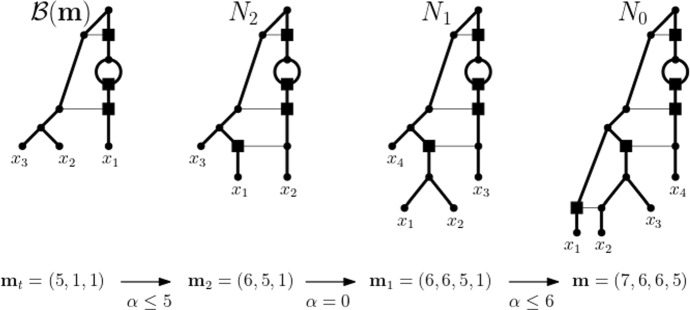
When reading from left to right, the construction of $N(\textbf{m})$ obtained from the traceback through the simplification sequence $\sigma (\textbf{m})$ for the ploidy profile $\textbf{m}=(7,6,6,5)$ on $\{x_1,\ldots , x_4\}$. The ploidy profiles that make up $\sigma (\textbf{m})$ are given at the bottom. The terminal element $\mathbf {m_t}$ of $\sigma (\textbf{m})$ is the ploidy profile (5, 1, 1), and the phylogenetic network ${\mathcal {B}}(\textbf{m})$ on the left is a core network for $\textbf{m}$. The cases that apply in each step of the traceback are indicated below the arrows between the four ploidy profiles that make up $\sigma (\textbf{m})$. The thin horizontal arcs relate to the example illustrating Theorem 1

To illustrate the construction of $N(\textbf{m})$, consider the ploidy profile $\textbf{m} = (7,6,6,5)$ on $X= \{x_1,x_2,x_3,x_4\}$. Then, the sequence $\textbf{m}$, (6, 6, 5, 1), (6, 5, 1) (5, 1, 1) is the simplification sequence $\sigma (\textbf{m})$ for $\textbf{m}$. Since $\mathbf {m_t} = (5,1,1)$, the phylogenetic network depicted on the left of Fig. 2 is a core network for $\textbf{m}$. In fact, it is the core network ${\mathcal {B}}(\textbf{m})$ for $\textbf{m}$ whose construction is described in the proof of Theorem 1. The network $N_0$ on the right of Fig. 2 is the network $N(\textbf{m})$ when initializing its construction with ${\mathcal {B}}(\textbf{m})$. It is obtained via the traceback of $\sigma (\textbf{m})$ by applying the cases indicated below the arrows. To be able to reuse the example to help illustrate Theorem 1, we represent one of the incoming arcs of each of the reticulation vertices of the networks that make up the figure as a thin, horizontal arc. Note that the leaf labels between the networks do not necessarily translate between the networks due to the applied renaming scheme for the elements of the indexing sets.

We conclude this section by remarking that by Huber and Maher (2022, Theorem 2), $N(\textbf{m})$ employs the minimum number of reticulation vertices to realize $\textbf{m}$ provided (i) ${\mathcal {B}}(\textbf{m})$ uses the minimum number of reticulation vertices to realize the terminal element of the simplification sequence of $\textbf{m}$, and (ii) the case $\alpha >m_2'$ is never executed when constructing $N(\textbf{m})$ from ${\mathcal {B}}(\textbf{m})$ where $\alpha$ and $m_2'$ are as in the description of that case (see Figures 6 and 10 in Huber and Maher 2022 for more on this and the next section for an example).

## Comparing Realizations of One and the Same Ploidy Profile

As indicated in the previous section, a ploidy profile $\textbf{m}$ might have more than one core network. This immediately begs the question of how different realizations of a ploidy profile might be. For phylogenetic trees and, more recently, for general rooted (beadless) or unrooted phylogenetic networks, this type of question has generally been addressed in the form of understanding their space. From a formal point of view, this space which is called *tree space* in the case of phylogenetic trees and *network space* in the case of rooted (beadless) or unrooted phylogenetic networks is a graph. Calling that graph *G*, then the vertices of *G* are the phylogenetic trees or networks of interest and any two vertices of *G* are joined by an edge if one can be transformed into the other using some graph-editing operation such as the *Subtree Prune and Regraft operation (SPR)* for phylogenetic trees (Semple and Steel 2003) or one the operations described in Erdös et al. (2021), Huber et al. (2016), Janssen (2021) and van Iersel et al. (2022).

None of the operations described in those papers however preserve, in general, our central requirement that a network is a realization of a ploidy profile. For technical reasons which will allow us to extend the idea of tree/network space to a space of ploidy profiles, we first need to extend the notion of a phylogenetic network. To this end, we call a phylogenetic network where different leaves are allowed to share the same label a *multiple-labelled network*. In the form of, for example, multiple-labelled trees such structures have already been used successfully in a polyploidization context (Oxelman and Petri 2011; Rothfels 2021). For their usage in a more mathematical context, see e.g. Huber and Scholz (2020) and the references therein. For example, consider the phylogenetic network depicted in Fig. 4(ii). Then, the graph obtained as follows is a multiple-labelled network. First, remove the arc $$(w,s_4')$$ and one of the incoming arcs of $$h_6$$. Next, suppress $$h_6$$ and its parent and add two further vertices both of which we call $$x_1$$. Finally, add an arc $$(w,x_1)$$ that ends in one of the two new vertices $$x_1$$ and an arc $$ (s_4',x_1)$$ that ends in the other so that a cherry on the multiset $$\{x_1,x_1\}$$ is generated. Since the number of directed paths from the root of the resulting graph to each of its leaves is not affected by this process, we extend the definitions of a reticulation vertex and when a ploidy profile is realized by a phylogenetic network to multiple-labelled networks in the obvious way.

**Fig. 3:**
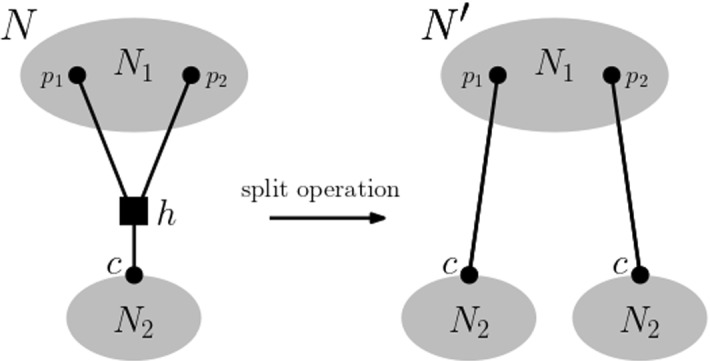
An illustration of the split operation applied to the reticulation vertex *h* with parents $p_1$ and $p_2$ and child *c*. $N_1$ and $N_2$ indicate parts of the multiple-labelled networks *N* and $N'$ that are of no relevance to the discussion

Armed with this, we are now ready to define ploidy profile space. Suppose $\textbf{m}$ is a ploidy profile on *X*. Then, we refer to the following graph as *ploidy profile space* ${\mathcal {P}}(\textbf{m})$ for $\textbf{m}$. The vertex set of the graph is the set of all multiple-labelled networks that realize $\textbf{m}$. To be able to define the edge set of our graph, we require a further concept. We say that a multiple-labelled network $N'$ is obtained from a multiple-labelled network *N* via a *split operation* if there exists a reticulation vertex *h* of *N* with parents $p_1$ and $p_2$ and child *c* such that (*h*, *c*) is a cut-arc and $N'$ is obtained from *N* as follows. First delete *h* and its three incident arcs from *N* and then make a copy of the subgraph of *N* induced by the vertices of *N* below *c*. Finally, add the arc $(p_2,c)$ as the incoming arc to one of the two copies of *c* and $(p_1,c)$ as the incoming arc of the other. We illustrate this operation in Fig. 3.

Informally speaking, the split operation may be thought of as “un-zipping” a multiple-labelled network (see also Pardi and Scornavacca 2015 for a related notion of “unzipping” a phylogenetic network). Choose an edit distance for comparing multiple-labelled trees that realize $\textbf{m}$ such that the following graph is connected. The vertex set is the set of all multiple-labelled trees that realize $\textbf{m}$ and any two multiple-labelled trees in that set are joined by an edge if their distance under the chosen edit distance is 1. For our next result (Proposition 1), we are interested in edit distances for which this space is connected (see Huber et al. 2011 for examples of such distances and also Lafond et al. 2019 for some recent computational complexity results concerning them).

Armed with the split operation and choice of edit distance, we continue our definition of ploidy profile space for $\textbf{m}$ as follows. We say that two distinct realizations *N* and $N'$ of $\textbf{m}$ are joined by an edge if either $N'$ can be obtained from *N* via a single split operation or *N* and $N'$ are both multiple-labelled trees and their distance under the chosen edit distance is 1.

### Proposition 1

For any ploidy profile $\textbf{m}$ on *X* and any edit distance on the set of multiple-labelled trees realizing $\textbf{m}$ such that the associated space of multiple-labelled trees is connected, ploidy profile space ${\mathcal {P}}(\textbf{m})$ is connected.

### Proof

Choose an edit distance *D* such that the associated space of multiple-labelled trees realizing $\textbf{m}$ is connected. Clearly, any realization *N* of $\textbf{m}$ can be transformed into a realization $N^+$ of $\textbf{m}$ that does not contain reticulation vertices that are above each other using a sequence of split operations. Since the vertex set of ploidy profile space is the set of multiple-labelled phylogenetic networks that realize $\textbf{m}$, it follows that $N^+$ can be transformed into a multiple-labelled tree that realizes $\textbf{m}$ by using a further sequence of split operations. By assumption on *D*, any multiple-labelled tree that realizes $\textbf{m}$ can be transformed into another multiple-labelled tree which also realizes $\textbf{m}$ via a sequence $\chi$ of multiple-labelled trees that all realize $\textbf{m}$, such that any two consecutive multiple-labelled trees in $\chi$ have distance 1 under *D*. Hence, ${\mathcal {P}}(\textbf{m})$ is connected. $\square$

As an immediate consequence of Proposition 1, we obtain a distance measure for realizations of a ploidy profile $\textbf{m}$. More precisely, choose an edit distance *D* for comparing two multiple-labelled trees that realize $\textbf{m}$ such that the space associated to *D* is connected. Then, we define the distance $D_{{\mathcal {P}}(\textbf{m})}(N,N')$ of any two realizations *N* and $N'$ of $\textbf{m}$ to be the length of a shortest path in ${\mathcal {P}}(\textbf{m})$ that joins *N* and $N'$. We refer the interested reader to Sect. 7 for an example where we compute an upper bound on this distance for a real biological dataset.

## A Core Network with Horizontal Arcs

Although undoubtedly useful, phylogenetic networks on their own do not provide information as to whether or not two species suspected of hybridization have existed at the same point in time. To add this type of realism to phylogenetic networks, so-called time stamp maps can be used. Subject to some constraints such maps assign a non-negative real number to every vertex of a phylogenetic network (see e.g. Baroni and Steel 2006; Francis and Steel 2015; van Iersel et al. 2021). As is well-known, not every phylogenetic network admits a time stamp map. However, those that do enjoy the attractive property that arcs whose both end vertices have been assigned the same time stamp can be drawn horizontally to indicate that the ancestral species represented by their end vertices have existed at the same time.

To be able to extend the notion of a time stamp map to ploidy profiles, we start with the definition of a certain time stamp map for (beadless) phylogenetic networks that originally appeared in van Iersel et al. (2021). Suppose that *N* is a beadless phylogenetic network on a set *X* with at least two elements. Then, a map $t:V(N)\rightarrow {\mathbb {R}}_{\ge 0}$ is called a *HGT-consistent labelling* of *N* if the following properties hold:

1. For all arcs (*u*, *v*) of *N*, we have that $t(u)\le t(v)$ if *v* is a reticulation vertex and, otherwise, that $t(u)<t(v)$.
2. For each vertex *u* that is not a leaf of *N*, there exists a child *v* such that $t(u)<t(v)$.
3. For each reticulation vertex *v* of *N*, there exists precisely one parent *u* such that $t(v)=t(u)$.

Informally speaking, Property (P1) means that time is moving forward, from the root of the network to its leaves. Property (P2) implies that every ancestral species *v* has given rise to at least one species that did not exist at the same time as *v*. Finally, Property (P3) implies for every reticulation vertex *v* that the ancestral species represented by *u* must have existed at the same time as the species represented by *v*. Examples of (beadless) phylogenetic networks that admit a HGT-consistent labelling include *stackfree* phylogenetic networks, that is, (beadless) phylogenetic networks that have no arcs for which both end vertices are reticulation vertices (see Bai et al. 2021 for more on such networks). It should however be noted that there exist (beadless) phylogenetic networks that admit such a labelling which might not be stackfree.

Since the definition of a HGT-consistent labelling of a beadless phylogenetic network *N* does not rely on the assumption that *N* has no beads, we extend it to our type of phylogenetic network by dropping the “beadless” requirement and qualifying Property (P3) by excluding reticulation vertices that are contained in beads. In combination, Property (P2) and the thus adjusted Property (P3) imply that there cannot have existed an ancestral species *v* such that *v* is involved in a polyploidization event and, at a later point in time, one of its parents, *p* say, hybridizes with the unique child *u* of *v*. Put differently, we cannot simultaneously have all three arcs (*v*, *u*), (*p*, *v*), and (*p*, *u*) and *v* is a reticulation vertex.

In view of the aforementioned combined effect of Properties (P2) and (P3), we also say that a phylogenetic network *N* admits a *weak HGT-consistent labelling* *t* if *t* is a map from the vertex set of *N* to the set of non-negative real numbers such that Properties (P1) and (P2) hold and Property (P3) is weakened to

(P3’) there exists at most one reticulation vertex *v* with distinct parents $u_1$ and $u_2$ with $u_2$ below $u_1$ such that *v* does not satisfy Property (P3), i.e. $t(u_i)\not =t(v)$, for all $i=1,2$.

As a first observation, note that a HGT-consistent labelling of a phylogenetic network is also a weak HGT-consistent labelling for that network.

To be able to state Theorem 1 which is concerned with clarifying the structure of ploidy profiles that admit a HGT-consistent labelling or a weak HGT-consistent labelling, we require the concept of a binary representation of a positive integer *m*. This representation essentially records for the representation of *m* as a sum $\sum _{i=0}^l 2^q$ of “powers of two” the vector of exponents. More formally, we define the *binary representation* of a positive integer $m=\sum _{j=1}^k 2^{i_j}$ to be the vector $(i_1,\ldots , i_k)$, $k\ge 1$, with $i_{j-1}>i_j$, for all $2\le j\le k$, and $i_k\ge 0$. Note that although related, the binary representation of *m* is not the bit-wise representation of *m*. For example, for $m=77=2^6+2^3+2^2+2^0$, the binary representation is (6, 3, 2, 0), whereas the bit-wise representation is (1, 0, 0, 1, 1, 0, 1).

We say that a strictly simple ploidy profile $\textbf{m}=(m_1)$ is *arc-rich* if the dimension of the binary representation of $m_1$ is at least two. Furthermore, we call a ploidy profile $\textbf{m}$ *practical* if either $\textbf{m}$ is simple but not strictly simple or $\textbf{m}=(m_1)$ and $m_1$ is of the form $m_1=2^l$, some $l\ge 1$. For example, the ploidy profile $\textbf{m}=(77)$ is arc-rich but not practical since the binary representation of 77 is the vector (6, 3, 2, 0).

### Theorem 1

Suppose $\textbf{m}$ is a ploidy profile. If the terminal element $\mathbf {m_t}$ of the simplification sequence for $\textbf{m}$ is practical, then there exists a core network for $\textbf{m}$ that admits a HGT-consistent labelling. Otherwise, $\mathbf {m_t}$ is arc-rich and there exists a core network for $\textbf{m}$ that admits a weak HGT-consistent labelling.

### Proof

For ease of readability, we split the proof into three sections, as indicated below. We start with introducing a further concept. Suppose *T* is a phylogenetic tree on $X=\{x_1,\ldots , x_n\}$, some $n\ge 2$. Then, we call *T* a *caterpillar tree* (on *X*) if the elements of *X* can be relabelled in such a way that *T* has a single cherry and that cherry is $\{x_{n-1},x_n\}$. If $n\ge 3$, then $x_1$ is a leaf that is a child of the root $\rho$ of *T*, and every vertex on the path from $\rho$ to the shared parent *f* of $x_n$ and $x_{n-1}$ other than $\rho$ and *f* has a child that is a leaf. For ease of presentation, we assume that the other child of the parent $f'$ of *f* is $x_{n-2}$, the other child of the parent of $f'$ is $x_{n-3}$ and so on.

**Fig. 4:**
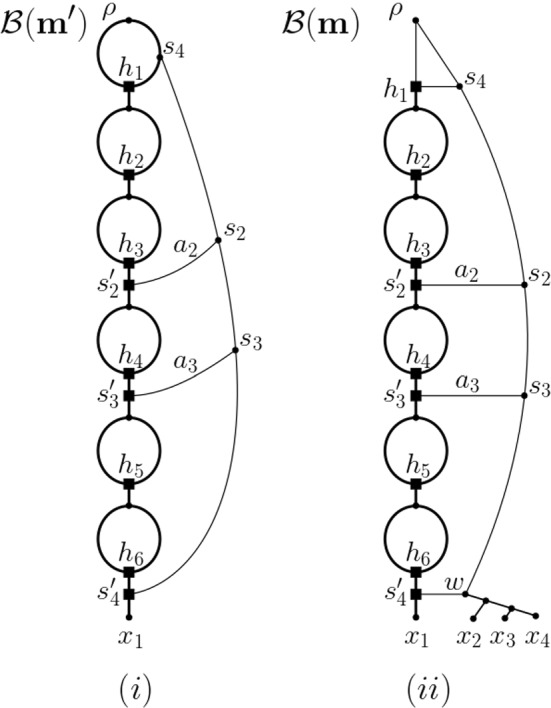
(i) Core network ${{\mathcal {B}}}(\mathbf {m'})$ for the strictly simple ploidy profile $\mathbf {m'}=(77)$ on $X=\{x_1\}$. (ii) The core network ${\mathcal {B}}(\textbf{m})$ for the simple ploidy profile $\textbf{m}=(77,1,1,1)$ on $\{x_1,x_2,x_3,x_4\}$ obtained from ${\mathcal {B}}(\mathbf {m'})$. Alternative core networks for $\textbf{m}$ can be obtained from ${\mathcal {B}}(\mathbf {m'})$ by subdividing non-bold arcs and attaching the remaining elements of *X* as phylogenetic trees on subsets of *X* or individually (ensuring that the arc $(s_3,s_4')$ is subdivided at a least once as otherwise the resulting phylogenetic network does not admit a HGT-consistent labelling because Property (P3) is violated)

For the remainder of the proof, assume that $\textbf{m}$ is a simple ploidy profile (see Fig. 4 for an illustration of our constructions for the ploidy profile $\textbf{m}=(77,1,1,1)$ on $X=\{x_1,\ldots , x_4\}$). Since a core network for $\textbf{m}$ realizes the terminal element $\mathbf {m_t}=(m_1,\ldots , m_n)$ of the simplification sequence for $\textbf{m}$ and $\mathbf {m_t}$ is simple, we need to consider the cases that $\mathbf {m_t}$ is strictly simple and that $\mathbf {m_t}$ is not strictly simple. Let $X=\{x_1,x_2,\ldots , x_n\}$ denote the set that indexes $\mathbf {m_t}$. Recall that, by convention, $x_i$ indexes $m_i$, for all $1\le i\le n$.

*Construction of the core network* ${\mathcal {B}}(\textbf{m})$ Assume first that $\mathbf {m_t}$ is a strictly simple ploidy profile. Then, $\mathbf {m_t}=(m_1)$ and $X=\{x_1\}$. Let $\textbf{i}=(i_1,\ldots , i_k)$, some $k\ge 1$, denote the binary representation of $m_1$. Note that $i_1\ge 1$ because $m_1\ge 2$. Then, we first construct a beaded tree $B(i_1)$ that realizes the strictly simple ploidy profile $(i_1)$ by taking $i_1$ beads $B_1, B_2,\ldots B_{i_1}$ and, provided $i_1\ge 2$, adding for all $1\le i\le i_1-1$ an arc from the reticulation vertex $h_i$ of $B_i$ to the tree vertex of $B_{i+1}$. To the resulting graph, we then add the vertex $x_1$ and an arc $(h_{i_1},x_1)$ to obtain $B(i_1)$. If $m_1=2^l$, some positive integer *l*, then we define ${\mathcal {B}}(\textbf{m})$ to be $B(i_1)$.

So assume that there exists no positive integer *l* such that $m_1=2^l$. Then, $k\ge 2$. Let $B(i_1,i_k)$ denote the phylogenetic network obtained from $B(i_1)$ by subdividing one of the two outgoing arcs of the root $\rho$ of $B(i_1)$ by a subdivision vertex $s_k$, the outgoing arc of the reticulation vertex in $B(i_1)$ that has precisely $i_k$ reticulation vertices strictly below it by a vertex $s_k'$ and adding the arc $a_k=(s_k,s_k')$. If $k=2$, then ${\mathcal {B}}(\textbf{m})$ is $B(i_1,i_2)$.

Finally, assume that $k\ge 3$. Then, we first construct the graph $B(i_1,i_k)$. Next, we subdivide the arc $a_k$ by $k-2$ vertices $s_2,\ldots , s_{k-1}$ such that $(s_k,s_2)$ is an arc and $s_j$ is the parent of $s_{j+1}$ for all $2\le j \le k-2$. For all $2\le j\le k-1$, we next subdivide the outgoing arc of the reticulation vertex of $B(i_1,i_k)$ that has precisely $i_j$ reticulation vertices of $B(i_1)$ strictly below it by a vertex $s_j'$. Finally, we add for all such *j* the arc $a_j=(s_j, s_j')$ and denote the resulting graph by ${\mathcal {B}}(\textbf{m})$ in this case. By construction, ${\mathcal {B}}(\textbf{m})$ is a phylogenetic network on $x_1$ that realizes $\mathbf {m_t}$ in either of these cases for *k*.

So assume that $\mathbf {m_t}$ is not strictly simple. Then, $m_j=1$, for all $2\le j\le n$. Using the same notation as before, we first construct the network ${\mathcal {B}}(\mathbf {m'})$ for the ploidy profile $\mathbf {m'}=(m_1)$. If $k=1$, then there exists some positive integer *l* such that $m_1=2^l$. Hence, ${\mathcal {B}}(\textbf{m})$ is $B(i_1)$ and we subdivide one of the outgoing arcs of the root of $B(i_1)$ by a vertex *w*. So assume that $k\ge 2$. If $k=2$, then ${\mathcal {B}}(\mathbf {m'})$ is $B(i_1,i_2)$ and we subdivide the arc $a_2$ of ${\mathcal {B}}(\mathbf {m'})$ by a vertex *w*. So assume $k\ge 3$. Then, we subdivide the arc $a=(s_{k-1},s_k')$ of ${\mathcal {B}}(\mathbf {m'})$ by a vertex *w*. In either of these cases for *k*, we then attach the caterpillar tree *T* on $\{x_2,\ldots , x_n\}$ to ${\mathcal {B}}(\mathbf {m'})$ via an arc from *w* to the root of *T* in case $n\ge 3$. If $n=2$, then we attach the vertex $x_n$ via the pendant arc $(w,x_n)$. By construction, the resulting graph is a phylogenetic network that realizes $\mathbf {m_t}$, and it is the network ${\mathcal {B}}(\textbf{m})$ in this final case for $\mathbf {m_t}$.

*Construction of a HGT-Consistent Labelling for* ${\mathcal {B}}(\textbf{m})$ *in Case* $\mathbf {m_t}$ *is Practical* Assume first that $\mathbf {m_t}$ is strictly simple. Then, $m_1=2^{i_1}$ and ${\mathcal {B}}(\textbf{m})$ is $B(i_1)$. Hence, there exists a directed path $P:v_0=\rho , v_1, v_2,\ldots , v_q=x_1$ from the root $\rho$ of ${\mathcal {B}}(\textbf{m})$ to $x_1$ once one arc has been removed from each bead of ${\mathcal {B}}(\textbf{m})$. Note that *P* contains vertices with indegree and outdegree one and that *V*(*P*) is the vertex set of ${\mathcal {B}}(\textbf{m})$. Consider the map $t:V(P)\rightarrow {\mathbb {R}}_{\ge 0}$ defined by putting $t(v_0)=0$ and $t(v_{j+1})= t(v_j)+1$, for all other $0\le j\le q-1$. By construction, it follows that *t* is a HGT-consistent labelling for ${\mathcal {B}}(\textbf{m})$ in this case.

So assume that $\mathbf {m_t}$ is not strictly simple. Then, $\mathbf {m_t}$ must be simple because it is the terminal element of the simplification sequence for $\textbf{m}$. Let $P:v_0=\rho , v_1, v_2,\ldots , v_q=x_1$ denote the directed path in ${\mathcal {B}}(\textbf{m})$ from $\rho$ to $x_1$ obtained by removing (i) the caterpillar tree on $\{x_2,\ldots , x_n\}$ and the incoming arc of its root in case $n\ge 3$ and $x_n$ and its pendant arc if $n=2$, (ii) for all $2\le j\le k$, the vertices $s_j$ and their incident arcs, and (iii) one of the two arcs in every bead. Let $t_P:V(P)\rightarrow {\mathbb {R}}_{\ge 0}$ be defined as the map *t* in the previous case.

Consider the map $t:V({\mathcal {B}}(\textbf{m}))\rightarrow {\mathbb {R}}_{\ge 0}$ defined by putting $t(v)=t_P(v)$ for all vertices *v* of ${\mathcal {B}}(\textbf{m})$ that are also vertices on *P*. So let *v* be a vertex in ${\mathcal {B}}(\textbf{m})$ that does not lie on *P*. If $v=s_k$, then put $t(v)=t(h_1)$, and if $v=w$, then put $t(v)=t(s'_k)$. For all $2\le j\le k-1$, put $t(s_j)= t(s'_j)$. Note that this does not violate Properties (P1)–(P3) since, for all $2\le j \le k-1$, we have $t(s_j')<t(s_{j+1}')$ and $t(s_k)<t(h_1)<t(s_2')=t(s_2)$. If $n\ge 3$, then for all $1\le j\le n-1$, let $w_j$ denote that parent of the leaf $x_{j+1}$ of *T*. Put $t(w_1)=t(w)+1$ and, for all $1\le j\le n-2$, put $t(w_{j+1})=t(w_j)+1$. Finally, choose a value $\chi >t(w_{n-1})$ and put $t(x_j)=\chi$, for all $2\le j\le n$. By construction, *t* respects Properties (P1)–(P3), and so *t* is a HGT-consistent labelling for ${\mathcal {B}}(\textbf{m})$. If $n=2$, then we proceed in a similar manner in that we put $t(x_n)=t(w)+1$.

*Construction of a Weak HGT-Consistent Labelling for* ${\mathcal {B}}(\textbf{m})$ *in Case* $\mathbf {m_t}$ *is Not Practical* If $\mathbf {m_t}$ is not practical it must be arc-rich as $\mathbf {m_t}$ is the terminal element of the simplification sequence of $\textbf{m}$. It suffices to note that a weak HGT-consistent labelling can be constructed as in the case of a HGT-consistent labelling noting that the only reticulation vertex of ${\mathcal {B}}(\textbf{m})$ that violates Property (P3) is $s_k'$. Thus, *t* satisfies Property (P3’) and so ${\mathcal {B}}(\textbf{m})$ admits a weak HGT-consistent labelling. $\square$

As mentioned in the proof of Theorem 1, we next illustrate the construction of ${\mathcal {B}}(\textbf{m})$ for the ploidy profile $\textbf{m}=(77,1,1,1)$ on $X=\{x_1,x_2,x_3,x_4\}$. Then, the vector $\textbf{i}=(6,3,2,0)$ is a binary representation for 77 and the phylogenetic network depicted in Fig. 4(i) is ${\mathcal {B}}(\mathbf {m'})$ where $\mathbf {m'}=(77)$. Clearly, the phylogenetic network ${\mathcal {B}}(\textbf{m})$ depicted in Fig. 4(ii) obtained from ${\mathcal {B}}(\mathbf {m'})$ by adding the leaves $x_2$, $x_3$, and $x_4$ as indicated realizes $\textbf{m}$ and admits a HGT-consistent labelling. Since the actual time stamp values are of no interest to our discussion, we indicate arcs for which both end vertices have the same time stamp under a HGT-consistent labelling in terms of horizontal arcs.

**Fig. 5:**
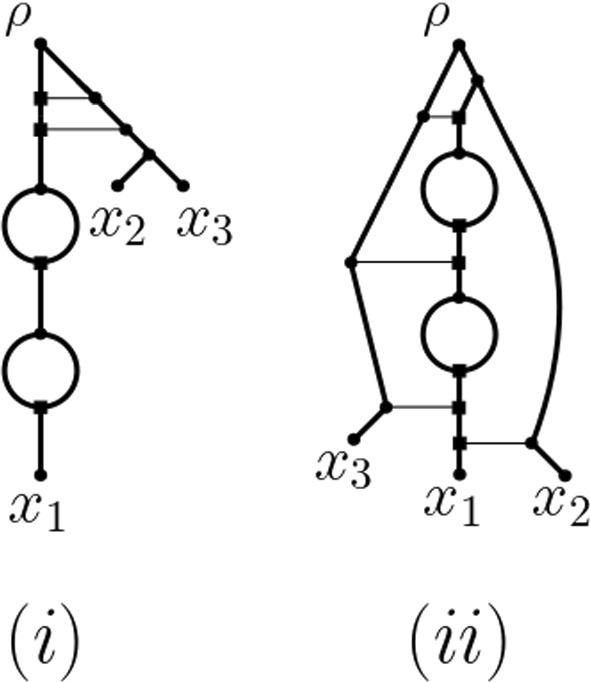
(i) Realization ${\mathcal {B}}(\textbf{m})$ of the practical ploidy profile $\textbf{m}=(12,1,1)$ on $X=\{x_1,x_2, x_3\}$. (ii) A core network for $\textbf{m}$ that is not of the form ${\mathcal {B}}(\textbf{m})$

As indicated in Fig. 5, alternative choices of a core network for a ploidy profile $\textbf{m}$ are conceivable in the sense that it might not be obtained by starting with a binary representation of the first component of $\textbf{m}$. Furthermore and perhaps not surprisingly, a core network *N* for $\textbf{m}$ generally admits more than one HGT-consistent labelling in the sense that an alternative HGT-consistent labelling for *N* might assign for a reticulation vertex *v* the same time stamp as for *v* to a different parent of *v*.

The fact that the simplification sequence of a ploidy profile $\textbf{m}$ is obtained by taking differences of the first two consecutive components of $\textbf{m}$ implies that, in general, properties of ploidy profiles do not get inherited by ploidy profiles obtained from $\textbf{m}$. For certain types of ploidy profiles, this is however not the case as the following consequence of Theorem 1 shows.

### Corollary 1

Suppose that $\textbf{m}$ is a ploidy profile. Then, the following holds.

(i) If $\textbf{m}$ is of the form $(n,n-1,n-2,\ldots , 1)$, $n\ge 3$, then, for any ploidy profile obtained from $\textbf{m}$ by removing at most one of its components, there exists a core network that admits a HGT-consistent labelling.
(ii) If $\textbf{m}=(m_1,\ldots , m_n)$, then there exists a core network for $(m_1,\ldots , m_n,1)$ that admits a HGT-consistent labelling.
(iii) If $\textbf{m}=(m_1,\ldots , m_n)$ has a core network that admits a HGT-consistent labelling, then the ploidy profile $(2^im_1, \ldots , 2^im_n,2^{i-1},2^{i-2}\ldots , 2^0)$, $i\ge 1$, has a realization that also admits such a labelling.

### Proof

(i) Let $\mathbf {m'}$ denote a ploidy profile obtained from $\textbf{m}$ as described in the statement of the corollary. Then, the difference between any two consecutive component values in $\textbf{m}$ is 1 if no component is removed from $\textbf{m}$ (i.e. $\textbf{m}=\mathbf {m'}$) or if a component is removed from $\textbf{m}$ to obtain $\mathbf {m'}$ whose value is not 2. In either of these two cases, it follows that the terminal element $\mathbf {m'_t}$ of the simplification sequence for $\mathbf {m'}$ is of the form $(2,1,\ldots , 1)$. If the component with value 2 is removed to obtain $\mathbf {m'}$ from $\textbf{m}$ then the terminal element $\mathbf {m'_t}$ of $\sigma (\mathbf {m'})$ is of the form $(3,1,\ldots , 1)$ as that ploidy profile is simple. In either of these cases, $\mathbf {m'_t}$ is practical. Applying Theorem 1 implies the result.

(ii) To see the assertion, it suffices to note that the terminal element of the simplification sequence for $\mathbf {m'}=(m_1,\ldots , m_n,1)$ is practical because it is of the form $(m,1, \ldots , 1)$, some $m\ge 2$.

(iii) Put $\mathbf {m'}=(2^im_1, \ldots , 2^im_n,2^{i-1},2^{i-2}\ldots , 2^0)$. Let ${\mathcal {B}}(\textbf{m})$ initialize the construction of $N=N(\textbf{m})$. Since, by assumption, ${\mathcal {B}}(\textbf{m})$ admits a HGT-consistent labelling, it follows by construction that *N* also admits such a labelling. Let $t:V(N)\rightarrow {\mathbb {R}}_{\ge 0 }$ denote a HGT-consistent labelling for *N*.

Next, consider the ploidy profile $\mathbf {m''}=(2^{i-1},2^{i-2}\ldots , 2^0)$ on *X* where $x_{n+j}$ indexes $2^{i-j}$, for all $1\le j\le i$. Then construct the core network ${\mathcal {B}}(\mathbf {m''})$ for $\mathbf {m''}$. Since $\mathbf {m''_t}=(2,1)$ and therefore is not strictly simple ${\mathcal {B}}(\mathbf {m''})$ admits a HGT-consistent labelling. Initializing the construction of $N''= N(\mathbf {m''})$ with ${\mathcal {B}}(\mathbf {m''})$ implies that $N''$ also admits a HGT-consistent labelling $t'':V(N'')\rightarrow {\mathbb {R}}_{\ge 0 }$.

Next, construct a realization $N'$ for $\mathbf {m'}$ from *N* and $N''$ by subdividing the incoming arc of $x_{n+1}$ by two new vertices *s* and $s'$ such that $s'$ is below *s*. Next, add a further vertex $s''$ and the arcs $(s,s'')$, $(s',s'')$, and $(s'',q)$ where *q* is the root of *N* to obtain $N'$. To obtain a HGT-consistent labelling $t':V(N')\rightarrow {\mathbb {R}}_{\ge 0}$ for $N'$ put $t'(v)=t''(v)$ for all vertices *v* of $N'$ that are also vertices in $N''$. Next, choose a value $t''(p)<\omega < t''(x_{n+1})$ where *p* is the parent of $x_{n+1}$ in $N''$ and put $t'(s)=\omega$, $t'(s')=t'(s'')=\omega +\epsilon$, some $\epsilon >0$ sufficiently small, and $t'(q)=t'(s)+1$. Finally, for all vertices *v* in *N*, put $t'(v)=t(v)+ t'(q)+1$. Since $t''$ is a HGT-consistent labelling for $N''$ and *t* is such a labelling for *N* it follows by construction that $t'$ is a HGT-consistent labelling for $N'$. $\square$

**Fig. 6:**
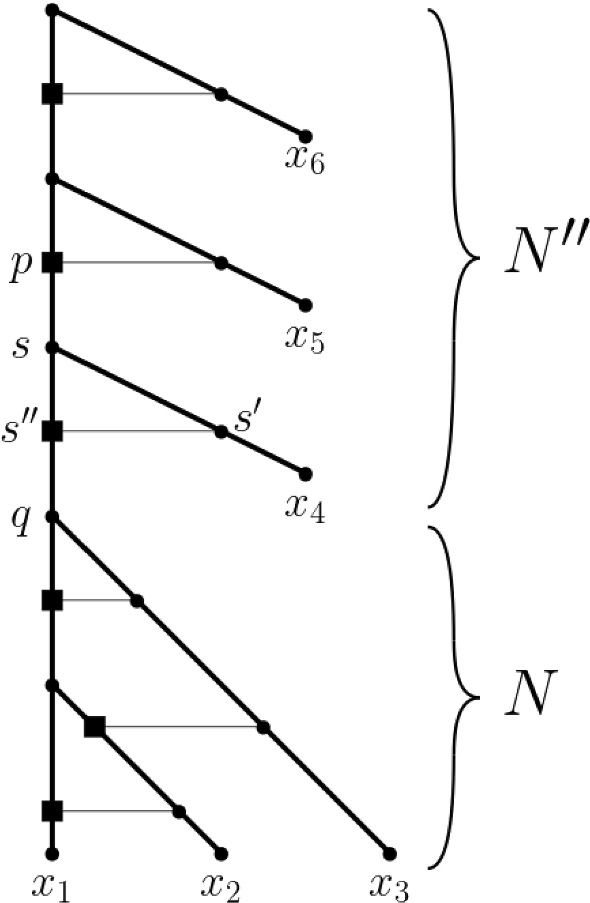
Realization $N'$ of the ploidy profile $\mathbf {m'}=(40,24,8,4,2,1)$ in terms of a phylogenetic network with horizontal arcs

To help illustrate Corollary 1(iii), consider the ploidy profile $\mathbf {m'}=(40,24,8,$ $4,2,1)=(2^3\times 5, 2^3\times 3, 2^3\times 1,2^2,2^1,2^0)$ on $X=\{x_1,\ldots , x_6\}$. Then, $i=3$ and $\textbf{m}$ is the ploidy profile (5, 3, 1) on $\{x_1,x_2,x_3\}$. Hence, $\mathbf {m_t}=(2,1,1)$. By Theorem 1, ${\mathcal {B}}(\textbf{m})$ admits a HGT-consistent labelling because $\mathbf {m_t}$ is practical. Initializing the construction of $N=N(\textbf{m})$ with ${\mathcal {B}}(\textbf{m})$ implies that *N* also admits a HGT-consistent labelling. The part of the network $N'$ pictured in Fig. 6 that is labelled *N* is that realization with a HGT-consistent labelling indicated in terms of horizontal arcs. The part of $N'$ labelled $N''=N(\mathbf {m''})$ is a realization of the ploidy profile $\mathbf {m''}=(4,2,1)$ on $\{x_4,x_5,x_6\}$ once the three incident arcs of $s''$ are ignored and $s''$ and the resulting vertices with indegree and outdegree one are suppressed. By construction, $N'$ is a realization of $\mathbf {m'}$ that admits a HGT-consistent labelling (again indicated in terms of horizontal arcs).

We conclude this section with remarking that Corollary 1(ii) is of particular interest from a “ghost species” point of view in that the element $x_{n+1}$ indexing the last component of $(m_1,\ldots , m_n,1)$ could represent a taxon with ploidy level one that has not been sampled yet (see e.g. Sardos et al. 2022 for the case of banana).

## When is $N(\textbf{m})$ a Tree with Additional Horizontal Arcs?

As was established in van Iersel et al. (2022, Section 2.1), beadless phylogenetic networks that admit a HGT-consistent labelling are precisely the ones that admit a so called complete cherry reduction sequence. These types of sequences essentially record how to transform a (beadless) phylogenetic network into a single vertex by applying only operations on pairs of leaves, provided this is possible. In view of van Iersel et al. (2022, Theorem 1) and Erdös et al. (2019), their attraction lies in the fact that they can be used to quickly check if a given (beadless) phylogenetic network can be represented with horizontal arcs without having to find a HGT-consistent labelling for it first. Therefore, it is of interest to see if an analogous result holds for our types of phylogenetic networks. To be able to shed light into this question, we first need to extend the concept of a cherry reduction sequence to our types of phylogenetic networks. For this, we require further terminology.

Suppose that *N* is a phylogenetic network on *X*. Assume first that *X* has at least two elements and that *a* and *b* are distinct elements in *X*. If $\{a,b\}$ is a cherry of *N*, then we refer to the operation of deleting *b* and its incoming arc and suppressing the resulting vertex of indegree and outdegree one as *reducing* the cherry $\{a,b\}$ by *b*. We denote this operation by ${\textit{reduce}}(a,b)$. Note that if the joint parent of *a* and of *b* is the root $\rho$ of *N* and *N* therefore has leaf set $\{a,b\}$, then this operation also includes post-processing the resulting graph by collapsing the unique arc from $\rho$ to *a* to obtain the single vertex *a*. If *a* and *b* form a reticulated cherry of *N* such that *b* is the reticulation leaf, then we refer to the operation of deleting the reticulation arc and suppressing the resulting vertices of indegree and outdegree one as *cutting* the cherry $\{a,b\}$. We denote this operation by *cut*(*a*, *b*). For example, deleting the thin arc incident with the parent of $x_1$ in the network $N_0$ pictured in Fig. 2 is the cutting operation $cut(x_2,x_1)$. Deleting the leaf $x_1$ in the network $N_1$ pictured in that figure is the reducing operation ${\textit{reduce}}(x_2,x_1)$.

Finally assume that *a* is the sole element of *X*. Then, we refer to the set $\{a\}$ as a *type-1 degenerate cherry* if the parent of *a* is the reticulation vertex in a bead *B*. In this case, we call the operation of removing one of the two arcs of *B*, suppressing resulting vertices with indegree and outdegree one, and also collapsing the unique outgoing arc of the tree vertex of *B* if that has rendered it a vertex of outdegree one as *simplification* of *a*. We denote this operation by ${\textit{simp}}(a)$. Furthermore, we refer to the set $\{a\}$ as a *type-2 degenerate cherry* if *a* has a parent *p* that is a reticulation vertex and either (i) precisely one of the parents $q_1$ and $q_2$ of *p* is the reticulation vertex of a bead or (ii) there exists a further vertex *q* such that *N* also contains (a) the three arcs $(q,q_1)$, $(q,q_2)$, and $(q_1,q_2)$, or (b) the arc $(q_1,q)$ and a pair of arcs from *q* to $q_2$. Assuming that Case (ii) holds then, we refer to the operation of deleting the arc $(q_1,p)$ (Case (a)) and deleting one of the arcs from *q* to $q_2$ (Case (b)) and in each case suppressing the two resulting vertices of indegree and outdegree 1 as *trimming* of *a*.

We denote this operation as ${\textit{trim}}(a)$. For example for the network pictured in Fig. 4(i), the trimming operation ${\textit{trim}}(x_1)$ consists of deleting the arc $(s_3,s_4')$ and suppressing the vertices $s_4'$ and $s_3$. Removing one of the two arcs in the bead in the network depicted in Fig. 5(i) that contains the parent of $x_1$ is the simplification operation ${\textit{simp}}(x_1)$.

It is easily seen that the operations of reducing a cherry and cutting a reticulated cherry both result in a phylogenetic network where, for technical reasons, we refer in this context to an isolated vertex *a* also as a phylogenetic network on $\{a\}$. Collectively, these two operations are usually referred to as *cherry reduction operations*. Since our type of phylogenetic networks may contain beads, we extend this convention by collectively referring to a cherry reduction operation, a simplification of a type-1 degenerate cherry, and the trimming of a type-2 degenerate cherry as a *cherry modification operation*.

Following Bai et al. (2021), we call a sequence $\chi$ of elements in *X* a *complete cherry reduction sequence* for a beadless phylogenetic network *N* on *X* if either (i) $\chi$ only contains *N* if *N* is a single vertex or (ii) $\chi$ is the sequence $N = N_0, N_1, N_2, \ldots , N_k, N_{k+1}$ of phylogenetic networks $N_i$, $0\le i\le k+1$, such that, for all $1\le i\le k+1$, the network $N_i$ is obtained from $N_{i-1}$ by a (single) cherry reduction operation and $N_{k+1}$ is a single vertex. A (beadless) phylogenetic network that admits a complete cherry reduction sequence is also called an *orchard*. With this in mind, we say that a phylogenetic network *N* of our type has a *complete cherry modification sequence* if *N* has an augmented complete cherry reduction sequence in the sense that, in addition to cherry reduction operations, the only other permitted operation is simplification of a type-1 degenerate cherry. For consistency reasons, we call a phylogenetic network that admits a complete cherry modification sequence also an *orchard* in this case.

Similarly, we call a sequence of cherry modifications operations a *complete weak cherry modification sequence* for *N*, if *N* has an augmented complete cherry modification sequence in the sense that, in addition to cherry reduction operations and the simplification of type-1 degenerate cherries, the trimming of a type-2 degenerate cherry is also allowed. In that case, we also call *N* a *weak orchard*.

**Fig. 7:**
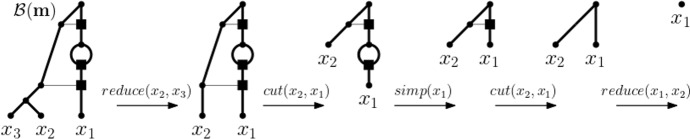
A complete cherry modification sequence for the core network ${\mathcal {B}}(\textbf{m})$ of $\textbf{m}=(7,6,6,5)$. The applied cherry modifications operations are indicated above the arrows between the networks

**Fig. 8:**
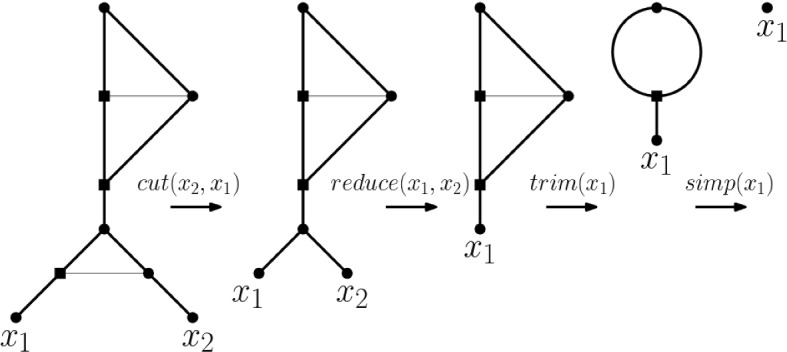
A weak cherry modification sequence for the realization $N(\textbf{m})$, depicted on the left, of the ploidy profile $\textbf{m}=(6,3)$ on $\{x_1,x_2\}$

For example and bearing in mind that the leaf labels are affected by the operations that govern the generation of the simplification sequence for $\textbf{m}$, the sequence of phylogenetic networks depicted in Fig. 2 read from right to left combined with the cherry modification sequence of the core network ${\mathcal {B}}(\textbf{m})$ of $\textbf{m}=(7,6,6,5)$ pictured in Fig. 7

is a complete cherry modification sequence for the realization $N(\textbf{m})$ of $\textbf{m}$ depicted in Fig. 2. On the other hand, the sequence presented in Fig. 8

is a weak cherry modification sequence for $N(\textbf{m})$ where $\textbf{m}$ is the ploidy profile (6, 3) on $\{x_1,x_2\}$.

Note that neither a complete cherry reduction sequence nor a complete weak cherry modification sequence might exist for a realization of a ploidy profile and also that, in case it does exist, such a realization might have more than one.

The fact that an orchard and also a weak orchard induces a ploidy profile by taking numbers of directed paths from the root of the network to each of its leaves lies at the heart of our extension of these concepts to ploidy profiles. More precisely, if $\textbf{m}$ is a ploidy profile that is realized by a phylogenetic network *N* and *N* is an orchard, then we call $\textbf{m}$ an *orchard (with respect to* *N*). If *N* is a weak orchard, then we call $\textbf{m}$ a *weak orchard (with respect to* *N*). To simplify terminology, we refer to $\textbf{m}$ simply as an orchard or a weak orchard if the knowledge of *N* is of no relevance to the discussion. For example and each time initializing the construction of the realization $N(\textbf{m})$ by ${\mathcal {B}}(\textbf{m})$, the ploidy profile $\textbf{m}=(7,6,6,5)$ is an orchard with respect to $N(\textbf{m})$, and the ploidy profile $\textbf{m}=(6,3)$ is a weak orchard with respect to its realization $N(\textbf{m})$. Thus, $\textbf{m}=(7,6,6,5)$ is an orchard and $\textbf{m}=(6,3)$ is a weak orchard. Furthermore, an exhaustive search for the ploidy profile $\textbf{m}=(3)$ shows that there exist ploidy profiles that are a weak orchard but not an orchard.

The next result formalizes a link suggested by these two examples between complete cherry modification sequences and simplification sequences. At its heart lies a characterization of (beadless) orchards in terms of HGT-consistent labellings (van Iersel et al. 2022, Theorem 1).

### Theorem 2

Suppose $\textbf{m}$ is a ploidy profile on *X*. If $\textbf{m}$ is practical then every ploidy profile in the simplification sequence $\sigma (\textbf{m})$ of $\textbf{m}$ is orchard. Furthermore, the traceback through $\sigma (\textbf{m})$ combined with a cherry modification sequence for ${\mathcal {B}}(\textbf{m})$ gives rise to a complete cherry modification sequence for $N(\textbf{m})$ provided the construction of $N(\textbf{m})$ is initialized with ${\mathcal {B}}(\textbf{m})$.

### Proof

Since $\textbf{m}$ is practical, Theorem 1 implies that ${\mathcal {B}}(\textbf{m})$ admits a HGT-consistent labelling. Combined with a canonical extension of van Iersel et al. (2022, Theorem 1) to our types of phylogenetic networks, it follows that there exists a complete cherry modification sequence for ${\mathcal {B}}(\textbf{m})$. To see that $N(\textbf{m})$ has a complete cherry modification sequence it therefore suffices to show that at each step in the traceback of $\sigma (\textbf{m})$ only a cherry or a reticulate cherry is introduced.

Assume for the remainder that the construction of $N(\textbf{m})$ is initialized with ${\mathcal {B}}(\textbf{m})$. Then, $N(\mathbf {m_t})$ has a complete cherry modification sequence by assumption on ${\mathcal {B}}(\textbf{m})$ as $N(\mathbf {m_t})$ is ${\mathcal {B}}(\textbf{m})$. Using the same notation as in the construction of $N(\textbf{m})$ outlined in Sect. 3 either $\alpha = 0$, or $\alpha >m_2'$, or $\alpha \le m_2'$. Let $N''$ denote a realization for $\mathbf {m''}$ constructed from ${\mathcal {B}}(\textbf{m})$ as described in the construction of $N(\textbf{m})$.

Employing the same indexing scheme as in the construction of $N(\textbf{m})$, it follows that to realize $\mathbf {m'}$, the leaf indexing the first component of $\mathbf {m''}$ is replaced by the cherry $\{x_1',x_2'\}$ if $\alpha =0$. In the two remaining cases, a single reticulate cherry on $\{x_1',x_2'\}$ with reticulate leaf $x_1'$ is generated. Thus, the generated realization of $\mathbf {m'}$, i. e.  $N(\mathbf {m'})$, is orchard. It follows that every ploidy profile in $\sigma (\textbf{m})$ is orchard. The remainder of the theorem is an immediate consequence. $\square$

Since, as mentioned in the proof of Theorem 2, the reversal of the operations to construct the network $N(\textbf{m})$ from the core network ${\mathcal {B}}(\textbf{m})$ corresponds to applying a single cherry reduction operation in each step of the traceback through $\sigma (\textbf{m})$, the companion result for ploidy profiles where ${\mathcal {B}}(\textbf{m})$ admits a weak HGT-consistent labelling also holds. Put differently, the result stated in Theorem 2 with the text “If $\textbf{m}$ is practical” omitted, the word “orchard”, replaced by “weak orchard” and the text “concatenated with a cherry modification sequence for ${\mathcal {B}}(\textbf{m})$ results in a complete cherry modification sequence for $N(\textbf{m})$” replaced by “concatenated with a weak cherry modification sequence for ${\mathcal {B}}(\textbf{m})$ results in a complete weak cherry modification sequence for $N(\textbf{m})$” also holds.

Intriguingly, the core network ${\mathcal {B}}(\textbf{m})$ for $\textbf{m}=(77,1,1,1)$ depicted in Fig. 4(i) gives rise to a phylogenetic tree on $\{x_1,\ldots ,x_4\}$ by deleting all horizontal arcs and removing one arc from each bead (each time suppressing the resulting vertices of indegree and outdegree one and the root in case this has rendered it a vertex with outdegree one). Beadless phylogenetic networks that enjoy this property are called tree-based (Francis and Steel 2015) and have recently attracted a considerable amount of attention in the phylogenetic networks community (see, for example, Steel 2016, Chapter 10.4.2) since they can be thought of as phylogenetic trees to which arcs have been added. More precisely, a phylogenetic network *N* is called *tree-based* if there exists a phylogenetic tree *T* such that when first adding an incoming arc to the root of *T* to obtain a tree $T'$ and then subdividing some of the arcs of $T'$ and adding arcs between the generated subdivision vertices (ensuring that no directed cycle is created and the overall degree sum of the subdivision vertices is 3) the resulting directed graph is *N*. In that case, *T* is called a *base tree* for *N*.

As it turns out, the above observation for $\textbf{m}=(77,1,1,1)$ and ${\mathcal {B}}(\textbf{m})$ is not a coincidence as the following more general result holds.

### Theorem 3

Suppose $\textbf{m}$ is a ploidy profile on *X*. If $\textbf{m}$ is practical, then the network $N(\textbf{m})$ generated from ${\mathcal {B}}(\textbf{m})$ is tree-based.

### Proof

This is an immediate consequence of Theorem 2 and the fact that the added horizontal arcs of $N(\textbf{m})$ correspond to reticulation arcs in reticulate cherries. $\square$

Interestingly, the corresponding result for arc-rich ploidy profiles does not hold as the core network depicted in Fig. 4(i) shows.

## A Viola Dataset

**Fig. 9:**
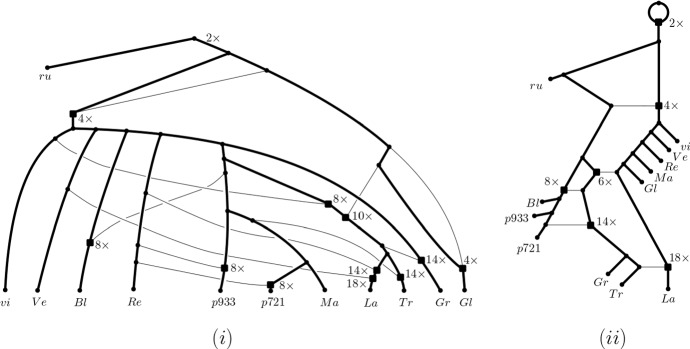
(i) A phylogenetic network on $X = \{$rubellium, viola, *V.verecunda*, *V.blande*, *V.repens*, *V.933palustris*, *V.721palustris*, *V.macloskeyi*, *V.langsdorff*, *V.tracheliffolia*, *V.grahamii*, *V.glabella*$\}$ adapted from Marcussen et al. (2012). To improve clarity, we include the ploidy level of each reticulation vertex. Apart from rubellium and viola which are denoted *ru* and *vi*, respectively, leaves are labelled by the first two characters of their name (omitting “*V.*”). (ii) The realization $N(\textbf{m})$ of the ploidy profile $\textbf{m}$ induced by the network in (i). Contrary to the network in (i), $N(\textbf{m})$ is orchard. In each case, the graph obtained by removing the non-bold arcs is a base tree for $N(\textbf{m})$. For ease of readability, the labels of the non-leaf vertices represent the number of directed paths from the root to that vertex in each of (i) and (ii)

In this section, we apply our findings to a simplified version of a dataset studied in Marcussen et al. (2012) to better understand the evolutionary past of plants in the genus Viola. The findings of the authors of that paper include a most parsimonious PADRE reconstruction of allopolyploid relationships within Viola, showing nine polyploidization events (two of which involve more than two ancestral species) to explain the dataset’s ploidy levels which range from $2\times$ to $18\times$ (Marcussen et al. (2012), Figure 4). To help ensure readability, we present a simplified version of that network in Fig. 9(i). To obtain it, we focused on (i) retaining the polyploidization events suggested by Marcussen et al. (2012, Figure 4) and the directed paths in the network which involve them, and (ii) representing subtrees in terms of single leaves. More precisely, we removed the taxa: *V.diffusa, V.papuana, V.selkirkii, V.somchetica, V.tuberifa, V.renifola, V.principis, V.vaginata, V.epipsila, V.pallens, V.lanceolata, V.primulifolia, V.jalapaënsis, V.occidentalis, V.pedata, V.clauseniana, V.sagittata, V.pubescens, V.lobata*. Furthermore, we summarized the taxa *V.capillaris* and *V.rubella* into the label rubellium as they formed a cherry and the taxa *V.laricicola, V.striata, V.stagnina, V.uliginosa, V.mirabilis, V.chelmea, V.collina, V.hirta* into the label viola as they formed a subtree. Finally, since the network in Marcussen et al. (2012, Figure 4) contained two vertices with indegree three, we have resolved them as indicated in Fig. 9(i). More precisely, the resolved vertices are the vertex labelled $10\times$ and its parent labelled $8\times$ and also the vertex labelled $18\times$ and its parent labelled $14\times$.

Although the network pictured in Fig. 9(i) clearly represents the ploidy profile $\textbf{m} = (18,14,14,10,8,8,8,4,4,2)$ by taking the number of directed paths from the root to each leaf, from a formal point of view, it is not a realization of $\textbf{m}$ since the ancestral species at the root is assumed to be diploid. This shortcoming of our framework can however easily be rectified by adding a bead via an incoming arc to the root of the network.

As was established in Huber and Maher (2022, Theorem 2), the minimum number of reticulation vertices required by a phylogenetic network to realize $\textbf{m}$ is 5. Since the phylogenetic network $N(\textbf{m})$ pictured in Fig. 9(ii) realizes $\textbf{m}$ using five reticulation vertices it follows that it is optimal with regard to this property. Furthermore, since none of the five reticulation vertices are contained in a bead, they all represent allopolyploidization events. Finally, $N(\textbf{m})$ admits a HGT-consistent labelling which implies that $\textbf{m}$ is orchard. In turn, this implies that a phylogenetic network that realizes $\textbf{m}$ can be obtained from a phylogenetic tree (in this case without beads) by adding horizontal arcs. Given these attractive features, it could be of interest to better understand to what extent the network $N(\textbf{m})$ can be used to inform the construction of a multiple-labelled tree such as the one underpinning the network in Fig. 9(i). As mentioned above already, constructing such a tree is not easy in general (Huber et al. 2009).

Using the insights from Sect. 4 to help assess how different the two networks in Fig. 9 are, assume that the chosen distance measure for comparing two multiple-labelled trees is the SPR-distance. Then by first applying split-operations to each of the two networks pictured in Fig. 9 until a multiple-labelled tree is obtained and then transforming one of the two obtained multiple-labelled trees into the other via a sequence $\chi _{SPR}$ of multiple-labelled trees such that two consecutive multiple-labelled trees in $\chi _{SPR}$ have SPR-distance 1 yields an upper bound of 26 on the $D_{{\mathcal {P}}(\textbf{m})}$-distance between the two networks.

## Concluding Remarks

In this paper, we have pushed back the current limits of the emerging field of *Polyploid Phylogenetics* (Rothfels 2021) by studying combinatorial properties of a ploidy profile $\textbf{m}$ on some set *X*. Denoting by $N(\textbf{m})$ the phylogenetic network obtained as a slightly modified version of the construction of a phylogenetic network that appeared in Huber and Maher (2022), we show that $N(\textbf{m})$ may be viewed as a generator of ploidy profile space ${\mathcal {P}}(\textbf{m})$ in the sense that any other realization *N* of $\textbf{m}$ can be obtained from it by going along the edges of a path from $N(\textbf{m})$ to *N* in ${\mathcal {P}}(\textbf{m})$ (Proposition 1). Furthermore, $N(\textbf{m})$ may be thought of as a phylogenetic tree with beads to which additional arcs have been added (Theorem 3) and at most one of these additional arcs cannot be drawn as a horizontal arc (Theorem 1). Furthermore, we establish a close link between the concept of a cherry reduction sequence for $N(\textbf{m})$ and the simplification sequence for $\textbf{m}$, a concept which underpins the construction of $N(\textbf{m})$ (Theorem 2). As an immediate consequence, we also have that the ploidy profile space for the ploidy profiles described in Corollary 1 can be generated from a phylogenetic tree with beads and only horizontal arcs added. Finally, we illustrate our findings by means of a real biological dataset.

Although our results are encouraging, numerous open questions remain. From a more biological point of view, these include understanding how well the $D_{{\mathcal {P}}(\textbf{m})}$-distance captures similarity between different realizations of a ploidy profile $\textbf{m}$. In the context of this, it should be noted that the edit-distance-type nature of the $D_{{\mathcal {P}}(\textbf{m})}$-distance implies that in general, it might be computationally difficult to compute it. This immediately begs the more mathematical question of how to bound it.

Also, it might be useful to explore diameter bounds for the $D_{{\mathcal {P}}(\textbf{m})}$-distance and the effect the choice of distance measure on multiple-labelled trees has. The same also holds when replacing the sequence of split operations to obtain a multiple-labelled tree from a phylogenetic network with the “unfold” operation for phylogenetic networks. Essentially, this operation associates a multiple-labelled tree to a phylogenetic network *N* by recording for every leaf *x* of *N* all directed paths from the root of *N* to *x* (see e.g. Huber and Moulton (2006); Huber et al. (2006) for details about this operation). It may also be interesting to explore the relationship between the simplification sequence and trinets (Huber and Moulton 2013). For example, how are the classes of phylogenetic networks that can be determined from trinets related to the class of phylogenetic networks with complete cherry reduction sequences?

In a different direction, it might be of interest to see if the results and approaches presented here can be extended to include further evolutionary processes such as aneuploidy whereby only a subset of the chromosome set of a genome (as opposed to the whole set of chromosomes) occurs multiple times. This could potentially involve representing a polyploid species not in terms of a ploidy level but in terms of a vector with each component representing the number of times the chromosome indexing it is found. A ploidy profile would in that case not be a vector of positive integers but a vector of vectors, each of them indexed by a species. Although attractive at first glance, this would require finding a new way of realizing a ploidy profile in terms of a phylogenetic network.

From a more mathematical point of view, it might also be interesting to investigate if a ploidy profile $\textbf{m}$ whose simplification sequences terminates in a practical ploidy profile can be characterized without having to compute the simplification sequence of $\textbf{m}$ first. This might require a better understanding of the link between simplifications sequences and ploidy profiles that are orchard. The availability of such a characterization could potentially lead to a fast way to decide if a ploidy profile $\textbf{m}$ can be realized by an orchard.

In the case of beadless phylogenetic networks, relationships between various types of properties are known. For example, every orchard is also tree-based (van Iersel et al. 2022, Corollary 2) and also every tree-child network is orchard (Bordewich and Semple 2016). Tree-child networks are defined as those (beadless) phylogenetic networks for which, for every one of its vertices *v*, there exists a directed path *P* to a leaf so that no vertex on *P* other than potentially *v* is a reticulation vertex. Extending this property canonically to our types of networks by allowing *P* to contain reticulation vertices in beads results in a natural way to extend the tree-child concept to our types of phylogenetic networks. More precisely, we call a ploidy profile *tree-child* if it has a realization that is tree-child when reticulation vertices in beads are ignored.

As is easy to see, if the construction of $N(\textbf{m})$ is initialized with the core network ${\mathcal {B}}(\textbf{m})$, then any ploidy profile of the form $(2^n,2^{n-1},\ldots ,2^1,2^0)$ with $n\ge 1$ is tree-child. However, at the same time, an exhaustive search for the ploidy profile $\textbf{m}=(3)$ shows that not every ploidy profile is tree-child. It might therefore be interesting to characterize tree-child ploidy profiles. This might involve better understanding properties of the core network for $\textbf{m}$ with which the construction of $N(\textbf{m})$ is initialized (see Fig. 5 for two alternative choices of a core network of the ploidy profile (12, 1) one of which is ${\mathcal {B}}(\textbf{m})$ and the other is not of the form ${\mathcal {B}}(\textbf{m})$). As part of this, it might be tempting to first focus on core networks obtained from a prime factor decomposition of the single component of a strictly simple ploidy profile (see also Huber and Maher 2022 for more on this).

## Data Availability

Apart from data already publicly available (see Marcussen et al. 2012), the manuscript has no data associated to it.

## References

[CR1] Albertin W, Marullo P (2012). Polyploidy in fungi: evolution after whole-genome duplication. Proc R Soc B.

[CR2] Bai A, Erdös PL, Semple C, Steel M (2021). Defining phylogenetic networks using ancestral profiles. Math Biosci.

[CR3] Baroni M, Steel M (2006). Hybrids in real time. Syst Biol.

[CR4] Bordewich M, Semple C (2016). Determining phylogenetic networks from inter-taxa distances. J Math Biol.

[CR5] Doyle JJ, Sherman-Broyles S (2017). Double trouble: taxonomy and definitions of polyploidy. New Phytol.

[CR6] Erdös PL, Semple C, Steel M (2019). A class of phylogenetic networks reconstructable from ancestral profiles. Math Biosci.

[CR7] Erdös PL, Francis A, Mezei TR (2021). Rooted NNI moves and distance-1 tail moves on tree-based phylogenetic networks. Discret Appl Math.

[CR8] Francis A, Steel M (2015). Which phylogenetic networks are merely trees with additional arcs?. Syst Biol.

[CR9] Huber KT, Maher LJ (2022) The hybrid number of a ploidy profile. J Math Biol 85:30 10.1007/s00285-022-01792-6 PMC9481518 36114394

[CR10] Huber KT, Moulton V (2006). Phylogenetic networks from multi-labelled trees. J Math Biol.

[CR11] Huber KT, Moulton V (2013). Encoding and constructing 1-nested phylogenetic networks with trinets. Algorithmica.

[CR12] Huber KT, Scholz GE (2020). Phylogenetic networks that are their own fold-ups. Adv Appl Math.

[CR13] Huber KT, Oxelman B, Lott M, Moulton V (2006). Reconstructing the evolutionary history of polyploids from multilabeled trees. Mol Biol Evol.

[CR14] Huber KT, Lott M, Moulton V, Spillner A (2009). The complexity of deriving a multi-labeled trees from bipartitions. J Comput Biol.

[CR15] Huber KT, Spillner A, Suchecki R, Moulton V (2011). Metrics on multilevelled trees: interrelationships and diameter bounds. IEEE/ACM Trans Comput Biol Bioinform.

[CR16] Huber KT, Moulton V, Wu T (2016). Transforming phylogenetic networks: moving beyond tree space. J Theor Biol.

[CR17] Huber KT, Linz S, Moulton V (2021) The rigid hybrid number of two phylogenetic trees. J Math Biol 82(5) 10.1007/s00285-021-01594-2 PMC7997861 33770290

[CR18] Janssen R (2021). Heading in the right direction? using head moves to traverse phylogenetic network space. J Graph Algorithms Appl.

[CR19] Janssen R, Murakami Y (2021). On cherry-picking and network containment. Theor Comput Sci.

[CR20] Jones G, Sagitov S, Oxelman B (2013). Statistical inference of allopolyploid species networks in the presence of incomplete lineage sorting. Syst Biol.

[CR21] Lafond M, El-Mabrouk N, Huber KT, Moulton V (2019). The complexity of comparing multiply-labelled trees by extending phylogenetic-tree metric. Theor Comput Sci.

[CR22] Leggatt RA, Iwama GK (2003). Occurrence of polyploidy in the fishes. Rev Fish Biol Fish.

[CR23] Lott M, Spillner A, Huber KT, Moulton V (2009). PADRE: a package for analysing and displaying reticulate evolution. Bioinformatics.

[CR24] Marcussen T, Jakobsen KS, Danihelka J, Ballard HE, Blaxland K, Brysting AK, Oxelman B (2012). Inferring species networks from gene trees in high-polyploid North American and Hawaiian violets (viola, violaceae). Syst Biol.

[CR25] Marcussen T, Sandve SR, Heire L, Spannagle M, Pfeiffer M, The international Wheat Genome Sequencing Consortium, Jakobsen KS, Wulff BBH, Steuernagel B, Mayer KF, Olsen A-A (2014) Ancient hybridizations among the ancestral genomes of bread wheat. Science 345 10.1126/science.1250092 25035499

[CR26] Oldman J, Wu T, van Iersel L, Moulton V (2021). Trilonet: piecing together small networks to reconstruct reticulate evolutionary histories. Mol Biol Evol.

[CR27] Oxelman B, Petri A (2011). Phylogenetic relationships within silene (Caryophyllaceae) section physolychnis. Taxon.

[CR28] Pardi F, Scornavacca C (2015). Reconstructible phylogenetic networks: do not distinguish the indistinguishable. PLoS Comput Biol.

[CR29] Rothfels CJ (2021). Polyploid phylogenetics. New Phytol.

[CR30] Sardos J, Breton C, Perrier X, Van den Houwe I, Carpentier S, Paofa J, Rouard M, Roux N (2022) Hybridization, missing wild ancestors and the domestication of cultivated diploid bananas. Front Plant Sci 13:969220 10.3389/fpls.2022.969220 PMC9586208 36275535

[CR31] Semple C, Steel M (2003). Phylogenetics.

[CR32] Semple C, Toft G (2021) Trinets encode orchard phylogenetic networks. J Math Biol 83:Article number: 28 10.1007/s00285-021-01654-7 34420100

[CR33] Steel M (2016) Phylogeny: discrete and random processes in evolution. SIAM

[CR34] The Potato Sequencing Consortium (2011) Genome sequence and analysis of the tuber crop potato. Nature 475:189–195 10.1038/nature10158 21743474

[CR35] van Iersel L, Janssen R, Jones M, Murakami Y, Zeh N (2018) Polynomial-time algorithms for phylogenetic inference problems. In: International conference on algorithms for computational biology. Springer, Berlin, pp 37–49 10.1109/TCBB.2019.2934957 31425045

[CR36] van Iersel L, Janssen R, Jones M, Murakami Y, Zeh N (2021). A unifying characterization of tree-based networks and orchard networks using cherry covers. Adv Appl Math.

[CR37] van Iersel L, Janssen R, Jones M, Murakami Y (2022) Orchard networks are trees with additional horizontal arcs. Bull Math Biol 84 10.1007/s11538-022-01037-z PMC9213324 35727410

[CR38] Vaoquaux F, Blanvillain R, Delseny P, Gallois P (2000). Less is better: new approaches for seedless fruit production. Trends Biotechnol.

